# Gastrointestinal and Hepatobiliary Complications of Extensively Drug-Resistant Typhoid at a Tertiary Care Hospital in Pakistan

**DOI:** 10.7759/cureus.11055

**Published:** 2020-10-20

**Authors:** Hala Mansoor, Khalil Ahmed, Samina Fida, Muhammad Uzair, Asma Asghar, Javed Iqbal

**Affiliations:** 1 Medicine, CMH Lahore Medical College and Institute of Dentistry, Lahore, PAK; 2 Surgery, Al-Aleem Medical College, Lahore, PAK

**Keywords:** pakistan, typhoid fever, xdr, gastrointestinal, hepatobiliary

## Abstract

Introduction: Typhoid fever is a major health problem in developing countries. Extensively drug-resistant (XDR) typhoid is an emerging threat to world health. The objectives of this study were to report our blood culture proven patients having XDR typhoid and compare the rate of gastrointestinal (GI) and hepatobiliary manifestations and complications of antimicrobial sensitive and resistant strains.

Materials and methods: This prospective observational study was carried out at a tertiary care hospital in Pakistan, from January 2019 till August 2020 on all consecutive blood culture proven patients of *Salmonella typhi*. A total of 57 cases of Salmonella were identified, of which 10 were nonresistant, seven multi drug-resistant (MDR), 39 extensively drug-resistant (XDR), and one was extended-spectrum beta lactamase (ESBL) positive. Alarmingly, one of the *S. typhi* isolate in addition to the first line drugs, was also resistant to azithromycin. Patients were treated with antibiotics according to antimicrobial susceptibility of the Salmonella in accordance with the World Health Organization (WHO) and Medical Microbiology and Infectious Diseases Society of Pakistan (MMIDSP) guidelines and GI and hepatobiliary complications were recorded.

Results: Overall rate of complications was low. Some 10% (1/10) with nonresistant typhoid, 14% (1/7) with MDR, and 15% (6/39) of our patients with XDR typhoid fever had abdominal tenderness (p=0.95). None of the patients had GI bleeding, abdominal abscess, or peritonitis. Some 20% (2/10) patients with nonresistant typhoid, 29% (2/7) with MDR, and 18% (7/39) with XDR typhoid developed acute hepatitis, with greater than three times elevation of liver transaminases. There was no statistically significant difference in the occurrence of hepatitis between these groups (p=0.98). Interestingly, one of our patients with XDR typhoid also developed cholestatic hepatitis.

Conclusion: There is no significant difference in GI and hepatobiliary complications amongst antimicrobial sensitive and resistant strains of typhoid. However, emergence of resistant strains calls for focus on prevention and judicious use of antimicrobials.

## Introduction

Typhoid fever is caused by Gram negative bacteria, *Salmonella typhi* (*S. typhi*). It continues to be a major health problem in the developing countries with 21.6 million cases causing about 250,000 deaths annually [1]. Feco-oral transmission through contaminated food and water, makes it the major health challenge in under-developed and developing countries [2]. *S. typhi* is the causative organism in as many as 30% of bacterial hematogenous infections in Asia [3].

In 2016, an outbreak started from Sindh, Pakistan of a drug-resistant strain that has further spread to other parts of the country and so far, more than 5200 patients of this extensively drug-resistant (XDR) strain have been reported. This strain remains resistant to the standard first line and second line agents used for treatment of enteric fever, including fluoroquinolones [4-5]. Presence of H58 haplotype with qnrSfluoroquinolone resistance gene and blaCTX-M-15 gene with production of extended-spectrum beta-lactamase (ESBL) leave it only sensitive to macrolides and carbapenems [6]. The same strain of bacteria has been isolated from travelers in the United Kingdom and United States, returning from Pakistan [4]. Industrialized countries have eliminated typhoid fever by improving sanitation and drinking water. However, the spread of drug-resistant strains threatens the efficacy of antimicrobials with fear of resurgence of enteric fever across many countries in the world [7].

Antimicrobial resistance (AMR) is quite widespread, and patients who are treated with ineffective antimicrobials exhibit improper clinical response with excessive complications and high mortality. AMR also leads to prolonged fecal shedding of bacteria, which causes sustained spread in the community with emergence of secondary cases [8-10].

The aims of this study were to report our blood culture proven patients of XDR typhoid and compare the rate of gastrointestinal (GI) and hepatobiliary manifestations and complications of antimicrobial sensitive and resistant strains.

## Materials and methods

This prospective observational study was carried out at CMH Lahore Hospital, from January 2019 till August 2020 on all blood culture proven patients of *S. typhi*. All cultures were processed on Mueller-Hilton agar at 37°C, in a single laboratory and antibiotic susceptibility was assessed by disc diffusion technique and zones of inhibition were elucidated in accordance with the Clinical and Laboratory Standard Institute (CLSI) recommendations [11]. Minimal inhibitory concentration (MIC) break points for ceftriaxone and ciprofloxacin were ≥4 ug/mL, ≥8 for levofloxacin, and ≥32 ug/mL for azithromycin. Antimicrobial susceptibility was tested against ampicillin, chloramphenicol, trimethoprim-sulfamethoxazole, ceftriaxone, ciprofloxacin, azithromycin, and meropenem. ESBL presence was assessed by disc diffusion technique and phenotypic verification was done by using ceftazidime and cefotaxime alone as well as in combination with clavulanate.

The study was conducted in line with principles of Helsinki’s declaration, after getting approval from the institution’s ethics committee. All eligible patients who were admitted as medical inpatients with fever were followed. Only those patients in whom culture confirmed growth of Salmonella were included in the study. Participant’s demographic, clinical, and therapeutic details were recorded during the course of admission in a standardized proforma, after getting informed consent. Data were analyzed through Statistical Package for the Social Sciences (SPSS) version 25 for Windows. Quantitative variables like age, duration of fever, and laboratory test results were presented as mean with or without standard deviation. Qualitative variables were shown as frequencies and percentages. Linear regression analysis was used to find correlation between different parameters. Chi-square was used to probe the association of complications between drug sensitive, multi drug-resistant (MDR), XDR, and ESBL positive strains of Salmonella and value of p ≤ 0.05 was considered to be statistically significant.

Resistance patterns were assigned according to the World Health Organization (WHO) definitions, which were as follows [4].

*Nonresistant typhoid*: Typhoid fever caused by *S. typhi* and/or paratyphi A, B, or C strains which are sensitive to first line drugs (chloramphenicol, ampicillin, trimethoprim-sulfamethoxazole) and third generation cephalosporins, with or without resistance to second line agents (fluoroquinolones).

*Multi drug-resistant typhoid*: Defined as resistant to three first line drugs used to treat typhoid - chloramphenicol, ampicillin and trimethoprim-sulfamethoxazole, with or without resistance to second line agents.

*Extensively drug-resistant typhoid*: Defined as resistant to first and second line agents as well as third generation cephalosporins.

Medical Microbiology and Infectious Diseases Society of Pakistan (MMIDSP) has further added another category of ESBL positive typhoid based upon the culture isolates of Salmonella, which is defined as follows [12]:

*ESBL positive typhoid*: Typhoid fever caused by *S. typhi* strains which are resistant to third generation cephalosporins but may be sensitive to chloramphenicol, trimethoprim-sulfamethoxazole, or fluoroquinolones.

## Results

Out of the 57 study participants, 45 (79%) were male while the mean age of the patients was 30.7 years. Demographic and baseline characteristics of the patients are shown in Table 1.

**Table 1:** Demographics and baseline characteristics (n=57). SD, standard deviation

Mean age, years (range) ± SD	30.7 (15-55) ± 9.2
Gender, n (%)	
Male	45 (79)
Female	12(21)
Mean duration of fever, (SD)	13 ± 6.64
Fever (Fahrenheit), n (%)	
≥104	37 (65)
< 104	20 (35)
Prior antibiotics usage, n (%)	
Yes	13 (23)
No	44 (77)

**Figure 1:**
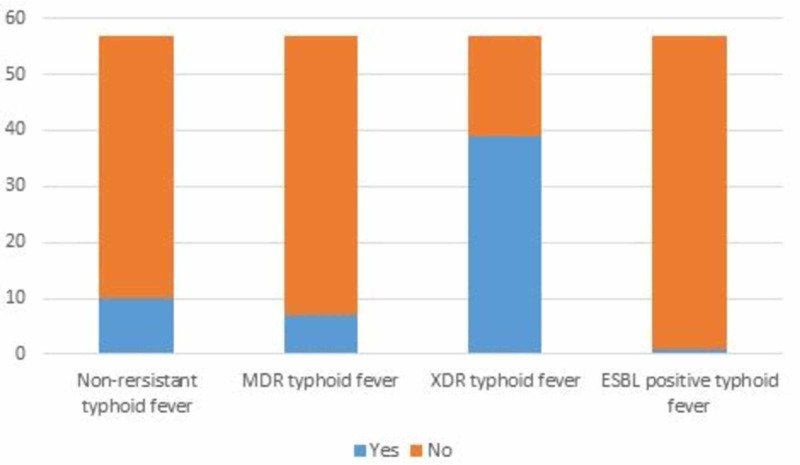
Resistance pattern of Salmonella isolates.

All patients had high grade fever on presentation with a mean duration of 13 ± 6.64 days. On admission, in all suspected patients of typhoid fever blood cultures were drawn and they were started on parenteral ceftriaxone 1 g every 12 hourly. In patients who did not show defervescence of fever on ceftriaxone after 48 hours, multiple further sets of blood as well as urine cultures were drawn. Once culture results were available, antimicrobials were switched according to the susceptibility results. All patients with XDR typhoid were treated with either alone or a combination of azithromycin and meropenem, according to MMIDSP guidelines [12]. The doses were calculated according to the body weight of the patients. Those who weighed <60 kg, were given a loading dose of 1 g of azithromycin, followed by 500 mg once daily, while those weighing >60 kg were given 1 g per day. Meropenem was given in a dose of 1000 mg every eight hourly. All patients completed 10-14 days of antibiotics. Of 57 *S. typhi* isolates 10 were nonresistant, seven MDR, 39 XDR and one was ESBL positive, as shown in the bar chart below (Figure 1). Fluoroquinolone resistance was quite widespread as 55 of 57 culture isolates were ciprofloxacin resistant and only two patients were sensitive. Alarmingly, one of the culture isolates in a 28-year-old male was also resistant to azithromycin in addition to resistance to all three first line antimicrobials. This isolate was sensitive to ciprofloxacin, ceftriaxone, and meropenem. A total of 13/57 patients had history of antibiotics intake for this fever prior to presenting in our hospital (2/10 patients with nonresistant typhoid, 1/7 with MDR, 9/39 with XDR, and 1/1 with ESBL positive typhoid fever, p= 0.29).

**Figure 2:**
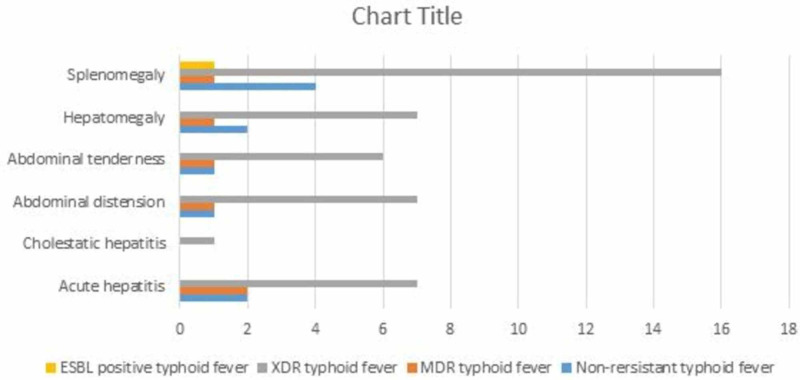
Gastrointestinal and hepatobiliary manifestations of typhoid fever.

Table 2 provides detail of clinical and laboratory parameters across various types of Salmonella based upon resistance. Linear regression analysis did not show any correlation between different laboratory and clinical parameters across various resistant types. Figure 2 demonstrates the GI manifestations and complications of typhoid fever in our patients. Two patients each in nonresistant and MDR, while seven patients in XDR group developed acute hepatitis, which was defined as three times elevation of aminotransferases from baseline. One male patient in XDR group developed acute right hypochondrial pain and cholestatic hepatitis during the course of admission in hospital. His serum bilirubin level increased to 70 umol/L, alanine aminotransferase (ALT) 220 IU/L, aspartate aminotransferase (AST) 210 IU/L, gamma-glutamyl transferase (GGT) 187 IU/L, and alkaline phosphatase (ALP) 1447 IU/L. Hepatitis serology for hepatitis A, B, C, and E was negative. Abdominal ultrasound was unremarkable and magnetic resonance cholangiography showed only intrahepatic cholestasis. He was treated with azithromycin and meropenem and made an uneventful recovery without any further complications. None of the patients in our series developed peritonitis, GI hemorrhage or gut perforation, neither there was any mortality. Comparison of GI complications amongst nonresistant, MDR, XDR and ESBL positive typhoid, did not show any statistically significant difference between these groups (p=0.98).

**Table 2:** Clinical and laboratory parameters across various susceptibility types. ALT, alanine aminotransferase; AST, aspartate aminotransferase; ALP, alkaline phosphatase; GGT, gamma-glutamyl transferase; SD, standard deviation

Parameters	Nonresistant typhoid	MDR typhoid	XDR typhoid	ESBL positive typhoid
Mean age in years (range)	31.4 (22-48)	29 (21-44)	31 (15-55)	33
Male gender, n (%)	8 (80)	4 (57)	33 (85)	0
Mean duration of fever in days ± SD	10.6 ± 5	13 ± 8	13.7 ± 7	8
Mean hemoglobin (g/dL) ± SD	14.1 ± 1.38	11.94 ± 1.18	12.8 ± 1.91	12.6
Mean WBC ± SD	4.6 ± 1.59	5.3 ± 1.41	7.35 ± 9.97	4.5
Neutrophil percentage ± SD	51.7 ± 15	58 ± 10.33	48.15 ± 13.71	32
Lymphocyte percentage ± SD	40 ± 14.71	34.29 ± 12.3	39.92 ± 12	59
Mean bilirubin (umol/L) ± SD	24.89 ± 24.69	16.86 ± 15.28	19.48 ± 20.93	19
Mean ALT (IU/L) ± SD	54 ± 47.96	86.43 ± 61.64	77.51 ± 71.05	46
Mean AST (IU/L) ± SD	48.4 ± 41.53	94.86 ± 74.15	87.54 ± 79.44	49
Mean ALP (IU/L) ± SD	179.9 ± 112.51	192.71 ± 108.70	280.77 ± 260.85	116
Mean GGT (IU/L) ± SD	72.60 ± 67.39	73.29 ± 28.11	93.84 ± 51.14	46
Mean albumin (g/dL) ± SD	3.8 ± 0.5	3.8 ± 0.78	82 (51.9)	4.2

## Discussion

Typhoid is one of the most common enteric infections of the developing countries. The organism’s feco-oral mode of transmission and predilection for the GI tract makes it the most common site of complications. As many as 30%-45% of patients report abdominal pain [13]. Association of fever with abdominal pain has 3.9 times higher odd of culture positive typhoid as compared to patients without abdominal pain [14]. Occasionally, the pain may be intense right iliac fossa, mimicking appendicitis or generalized abdominal as in peritonitis [15]. Constipation as well as diarrhea are common symptoms. While abdominal distension is also frequently noticed [14, 16]. Abdominal distension was noted in 1% (1/10) of our patients with nonresistant typhoid, 14.3% (1/7) with MDR typhoid, and in 18% (7/39) with XDR typhoid (p=0.89). While 10% (1/10) with nonresistant typhoid, 14% (1/7) with MDR, and 15% (6/39) of our patients with XDR typhoid fever had abdominal tenderness (p=0.95). None of the patients had severe tenderness to mimic peritonitis or appendicitis to warrant surgical evaluation. Involvement of Peyer’s patches of small intestine with hyperplasia, necrosis and ultimately bleeding, perforation and peritonitis in the third or fourth week of infection is one of the dreaded complications. Perforations can be multiple, require resection of entire segment and are associated with high mortality [13, 17-19]. Fortunately none of our patients suffered the harrowing complications of GI bleeding, abdominal abscess, or perforation.

Hepatomegaly is a common finding in patients with typhoid fever. It is associated with liver dysfunction, partly due to the effect of bacteria causing proliferation of the reticulo-endothelial cells and partly due to the effects of medications [20-21]. This manifests as variable elevation in serum bilirubin, liver transaminases, GGT or ALP. Picture is usually cholestatic, however, symptomatic cholestatic hepatitis is rare and there are only a few case reports [20, 22-23]. Hepatomegaly was seen in 2% (2/10) with nonresistant typhoid fever, 14% (1/7) with MDR typhoid, and 18% (7/39) of our patients with XDR typhoid (p=0.96). While 20% (2/10) patients with nonresistant typhoid, 29% (2/7) with MDR, and 18% (7/39) with XDR typhoid developed acute hepatitis, with greater than three times elevation of liver transaminases. There was no statistically significant difference in the occurrence of hepatitis between these groups (p=0.98). Interestingly, one of our patients with XDR typhoid developed cholestatic hepatitis, which as mentioned above is a rare entity. Although local inflammatory mediators, endotoxin as well as host immune response, have all variably been considered responsible for severe hepatic involvement in typhoid fever, the exact pathogenesis still remains to be ascertained. Possible risk factors for development of typhoid hepatitis include the virulence of S. typhi, poor functional status of the patient with comorbidities, and delayed start of treatment [24]. Typhoid hepatitis also has been suggested to increase the risk of relapse of typhoid fever [22]. The standard of care for cholestatic hepatitis, has been the administration of appropriate antibiotics according to the bacterial susceptibility [25]. Our patient with XDR typhoid, who developed cholestatic hepatitis was treated with meropenem and azithromycin and on follow up visit at six weeks, complete normalization of his liver function tests was noted.

Spleen being a reticuloendothelial organ, is frequently involved in typhoid fever. Splenic involvement can vary from splenomegaly which is very common, to splenic abscess or rupture that are infrequent complications [13, 26]. Splenomegaly was noted in 40% (4/10) of our patient with nonresistant typhoid, 14% (1/7) with MDR typhoid, 41% (16/39) with XDR typhoid, and 100% (1/1) of patients with ESBL positive typhoid fever (p=0.32). Fortunately none of our patients in any group, developed splenic abscess or rupture.

Other GI and abdominal complications that have been reported to be associated with typhoid fever include acalculus cholecystitis, Gram negative sepsis leading to gall bladder gangrene and perforation, concomitant gall bladder and ileal perforation, duodenal ulceration with polyserositis, ileal ulceration with hematochezia, abdominal abscess, ovarian abscess, pancreatitis leading to pancreatic abscess, mesenteric lymphadenitis, and ascites. None of these complications were observed in any of our patient with typhoid fever [13, 25, 27-30].

## Conclusions

The XDR typhoid fever is an emerging threat to public health around the world. GI tract and hepatobiliary complications have a major share in the spectrum of dreadful complications that can be encountered in these patients. Effective treatment of these complications requires antibiotic treatment according to the organism’s antimicrobial susceptibility. Finding of a bacterial isolate which was resistant to azithromycin is a cause of great concern, as the antimicrobial options for XDR typhoid are already limited. Although we did not find any statistical difference in the occurrence of GI or hepatobiliary complications in the antibiotic sensitive versus antibiotic resistant groups, the reliance on antibiotics for successful treatment calls for public and healthcare awareness for prevention and judicious use of antimicrobials to minimize the emergence and spread of resistant isolates.
